# Effects of computer-assisted navigation versus the conventional technique for total knee arthroplasty on levels of plasma thrombotic markers: a prospective study

**DOI:** 10.1186/s12938-019-0717-3

**Published:** 2019-10-14

**Authors:** Ka-Kit Siu, Kwan-Ting Wu, Jih-Yang Ko, Feng-Sheng Wang, Wen-Yi Chou, Ching-Jen Wang, Shu-Jui Kuo

**Affiliations:** 1 Department of Orthopedic Surgery, Kaohsiung Chang Gung Memorial Hospital, Kaohsiung, Taiwan; 2 Center for Shockwave Medicine and Tissue Engineering, Kaohsiung Chang Gung Memorial Hospital and Chang Gung University College of Medicine, Kaohsiung, Taiwan; 3 Department of Orthopedic Surgery, Xiamen Chang Gung Hospital, Xiamen, China; 4 School of Medicine, China Medical University, Taichung, Taiwan; 5 Department of Orthopedic Surgery, China Medical University Hospital, Taichung, Taiwan

**Keywords:** Navigation-assisted total knee arthroplasty, d-dimer, Fibrinogen

## Abstract

**Background:**

Venous thromboembolism (VTE) is a major sequela after total knee arthroplasty (TKA). We prospectively compared the differences in the perioperative plasma d-dimer and fibrinogen levels between the individuals undergoing TKA via computer-assisted navigation and via a conventional method as the surrogate comparison for VTE. There were 174 patients fulfilling the inclusion criteria and providing valid informed consent between September 2011 and November 2013. There were 69 females and 20 males in the navigation-assisted group (median age: 71.00 years), while the conventional group was composed of 59 females and 26 males (median age: 69.00 years). Blood samples were obtained prior to and at 24 and 72 h after surgery for measurement of the levels of plasma d-dimer and fibrinogen.

**Results:**

A significantly lower plasma d-dimer level 24 h after TKA (*p* = 0.001) and a milder postoperative surge 24 h after TKA (*p* = 0.002) were observed in patients undergoing navigation-assisted TKA. The proportions of subjects exceeding the plasma d-dimer cut-off values of 7.5, 8.6 and 10 mg/L 24 h after TKA were all significantly higher in the conventional group than in the navigation-assisted group (*p* = 0.024, 0.004, and 0.004, respectively).

**Conclusions:**

A lower plasma d-dimer level and a milder surge in the plasma d-dimer level were observed in patients undergoing navigation-assisted TKA in comparison with patients undergoing conventional TKA 24 h after surgery. These findings may supplement the known advantages of navigation-assisted TKA.

## Background

Osteoarthritis (OA) manifests as synovial inflammation, cartilage destruction, joint swelling and pain [1]. Total knee arthroplasty (TKA) is a well-established treatment modality with a high satisfaction rate for the treatment of end-stage knee OA [2]. Venous thromboembolism (VTE) after TKA has frequently been discussed in the literature [3–13]. The clinical presentation is variable, ranging from subclinical manifestation and deep vein thrombosis (DVT) to fatal pulmonary embolism (PE). The incidence of VTE after TKA has been reported to be 30–50% [7, 12–14]. More than half of VTE cases are asymptomatic [15]. Acquired risk factors for VTE include hip or knee joint replacement, major trauma, lower limb fractures and spinal cord injury [9]. The American Academy of Orthopedic Surgeons (AAOS) clinical guidelines for VTE published in 2011 suggested that patients undergoing elective hip or knee replacement surgery are already at high risk of VTE, even if they do not possess any other acquired or hereditary factors [10].

No single assessment is adequate for the diagnosis of DVT, mandating a combination of clinical evaluation, laboratory testing and imaging studies. Computed tomography (CT) angiography is the gold standard for the diagnosis of PE, and Doppler ultrasound or venography is commonly utilized for imaging validation of DVT [6, 9, 15, 16]. Clinical scoring systems for VTE prediction include Wells score and the revised Geneva score [5, 9]. Clinically, the plasma d-dimer level is used effectively to exclude VTE [17–20]; it is also used as an indicator of thromboembolic events [20].

Using navigation-assisted TKA, femur and tibia cutting can be performed accurately in an extramedullary manner, hence mitigating the destruction of the bone marrow cavity. A significant decrease in systemic emboli was observed by transesophageal echocardiography [21] and transcranial Doppler [22] soon after surgery in previous pilot studies. Our previous studies showed milder increases in serum endothelial injury markers and inflammation markers in a navigation-assisted TKA cohort as compared with a conventional TKA group [2, 23].

Based upon the findings mentioned above, we hypothesized that the limited violation of the distal femoral bone marrow cavity in navigation-assisted TKA might mitigate the postoperative surge in the levels of thromboembolic markers, which has been proposed as an indicator of thromboembolism. In this study, we prospectively compared the levels of perioperative plasma thrombotic markers between patients undergoing navigation-assisted and conventional TKA as the surrogate comparison of VTE. We also investigated the incidence of VTE or PE within 5 to 7 years after surgery.

## Results

A total of 174 patients who fulfilled the inclusion criteria and provided valid informed consent between September 2011 and November 2013 were recruited for the study. There were 69 females and 20 males in the navigation-assisted TKA group, with a median age of 71.00 years, while the conventional group was composed of 59 females and 26 males, with a median age of 69.00 years. There were no between-group differences in gender (*p* = 0.225), age (*p* = 0.980), operated side (*p* = 0.368), preoperative deformity (*p* = 0.916) or body mass index (*p* = 0.246). The proportions of subjects afflicted with diabetes (*p* = 0.992), hypertension (*p* = 0.879) and coronary artery disease (*p* = 0.696) were comparable between the two groups. A higher proportion of participants in the navigation-assisted group was afflicted with stroke before the surgery (*p* = 0.015) (Table 1).

**Table 1:** Demographic profiles between the navigation and conventional groups

	Navigation (*n* = 89)	Conventional (*n* = 85)	*p*-value
Gender	69 F, 20 M	59 F, 26 M	0.225
Age (years)	71.00 (63.00,75.00)	69.00 (64.00,75.00)	0.980
Operated side	49 left, 40 right	41 left, 44 right	0.368
Preoperative deformity	77 varus, 12 valgus	74 varus, 11 valgus	0.916
BMI (kg/m^2^)	27.48 (25.11, 29.59)	27.59 (25.78, 30.85)	0.246
Diabetes	21	20	0.992
Hypertension	64	62	0.879
CAD	5	6	0.696
Stroke	6	0	0.015

*F* female, *M* male, *BMI* body mass index, *CAD* coronary artery disease

At baseline, the plasma levels of d-dimer (*p* = 0.172) and fibrinogen (*p* = 0.847) were comparable between the two groups. The Friedman *p*-values were < 0.001 and < 0.001 for d-dimer and fibrinogen in the conventional group and < 0.001 and < 0.001 in the navigation-assisted group, respectively. The *p*-values of post hoc Wilcoxon signed-rank tests are shown in Tables 2, 3. Generally, the post-TKA plasma d-dimer and fibrinogen levels were all higher than those at baseline.

**Table 2:** Plasma d-dimer concentrations (mg/L) prior to TKA, then at 24 and 72 h after TKA

	Navigation	Conventional	*p*-value
Baseline	0.55 (0.35, 0.98)^ab^	0.56 (0.36, 0.99)^de^	0.172
24 h after TKA	4.60 (3.10, 8.83)^ac^	7.19 (3.89, 14.38)^df^	0.001
72 h after TKA	3.11 (2.32, 3.99)^bc^	2.70 (2.06, 4.10)^ef^	0.531
24 h-baseline	3.97 (2.42, 7.78)	6.76 (3.17, 13.88)	0.002
72 h-baseline	2.43 (1.47, 3.36)	2.16 (1.40, 3.24)	0.446

^a^*p* < 0.001; ^b^ *p* < 0.001; ^c^ *p* < 0.001; ^d^ *p* < 0.001; ^e^ *p* < 0.001; ^f^ *p* < 0.001 by Wilcoxon signed-rank test for post hoc analysis

**Table 3:** Plasma fibrinogen concentrations (mg/dL) prior to TKA, then at 24 and 72 h after TKA

	Navigation	Conventional	*p*-value
Baseline	282.00 (257.00, 330.00)^gh^	284.00 (248.50, 335.50)^jk^	0.847
24 h after TKA	292.00 (257.00, 323.00)^gi^	303.00 (259.00, 332.00)^jl^	0.325
72 h after TKA	523.00 (482.75, 589.25)^hi^	496.00 (469.00, 551.00)^kl^	0.232
24 h-baseline	− 6.50 (− 34.25, 23.00)	9.00 (− 17.00, 34.00)	0.175
72 h-baseline	238.00 (166.25, 298.00)	212.00 (169.00, 280.00)	0.360

^g^*p* = 0.099; ^h^*p* < 0.001; ^i^*p* < 0.001; ^j^*p* = 0.412; ^k^*p* < 0.001; ^l^*p* < 0.001 by Wilcoxon signed-rank test for post hoc analysis

At 24 h after surgery, the median plasma d-dimer level in the navigation-assisted group was 36.0% (*p* < 0.001), lower than that in the conventional group. The median postoperative increment in the plasma d-dimer level was 41.2% lower in the navigation-assisted group than in the conventional group; however, the between-group difference was diminished 72 h after surgery.

We also compared the proportions of subjects in whom various plasma d-dimer level cut-off values for VTE as described in the literature were exceeded [20, 24–26]. The proportions of subjects exceeding the cut-off values of 7.5 (*p* = 0.024), 8.6 (*p* = 0.004) and 10 mg/L (*p* = 0.004) 24 h after TKA were all significantly higher in the conventional group than in the navigation-assisted group (Table 4).

**Table 4:** Between-group differences of the proportion of subjects exceeding the various cut-off values of plasma d-dimer (mg/L) 24 h after surgery

d-dimer cut-off value	Navigation	Conventional	*p*-value
7.5	42	29	0.024
8.6	40	23	0.004
10	32	16	0.004

All of the participants were followed up for 5–7 years after surgery. None of the participants was readmitted due to acute postoperative complications such as hematoma, periprosthetic infection or fracture during the follow-up period. VTE was suspected in three patients in the conventional TKA group. The first patient was readmitted 1 month after surgery due to clinically suspected DVT (leg swelling and redness), with a periadmission plasma d-dimer level of 7.19 mg/L. Duplex scanning of the vein was arranged, and the result of which was negative. The second patient visited the cardiovascular surgery clinic for clinically suspected DVT (leg swelling and redness) 4 months after surgery. Her plasma d-dimer level was measured at 16.01 mg/L in the cardiovascular surgery clinic, and duplex scanning of the vein was arranged, with negative results. The third patient was referred to the Emergency Department 7 months after surgery (post-op d-dimer level: 18.25 mg/L) due to leg swelling and dyspnea. Under the impression of pulmonary embolism, chest CT was arranged, but no emboli were found (Table 5). The symptoms of these patients resolved after the administration of anticoagulants. None of the participants in the navigation-assisted TKA group visited the Emergency Department or the Outpatient Clinic due to symptoms of VTE.

**Table 5:** Summary of the suspicious VTE events within post-op 7 months follow-up

	d-dimer (mg/L)	Timing (months)	Presentation	Surveys
Case 1	7.19	1	Leg swelling, redness	Duplex: negative
Case 2	16.01	4	Leg swelling, redness	Duplex: negative
Case 3	18.25	7	Leg swelling, dyspnea	Chest CT: negative

All the three cases underwent conventional TKA

## Discussion

If not diagnosed and treated in a timely manner, the development of VTE after TKA may lead to a catastrophic outcome. Although certain regimens have been proposed for mechanical and pharmacological VTE prophylaxis, the ideal strategy for addressing VTE is to prevent rather than to treat. In our study, we attempted to demonstrate the reduced risk of VTE following TKA surgery using the reaming-free navigation technique by comparing proxy endpoints, consisting of the plasma d-dimer and fibrinogen levels, between a navigation-assisted TKA group and a conventional TKA group. We demonstrated a significantly lower plasma d-dimer level as well as a milder increase in the plasma d-dimer level 24 h after TKA in patients undergoing navigation-assisted TKA as compared with those undergoing conventional TKA. These results have not been published previously and are worthy of note.

d-dimer is the degradation product of fibrin that is formed immediately following the degradation of thrombin-generated fibrin clots, and the plasma concentration of d-dimer reflects the global activation of coagulation and fibrinolysis [18]. Other factors related to an elevated plasma d-dimer level include sepsis, inflammatory disease, malignancy, trauma, coagulation disorders and the aging process [17, 27]. The plasma d-dimer level is one of the most useful laboratory tests to supplement the diagnosis of VTE after TKA, and a normal plasma d-dimer safely excludes VTE [16]. Various cut-off values for the plasma D-dimer level for VTE evaluation have been reported in the literature. Watanabe et al. [20] showed that with a cut-off d-dimer value of 7.5 μg/mL, the sensitivity and specificity were 75% and 63% for VTE after TKA, respectively. For patients undergoing TKA with tourniquet use, the sensitivity, specificity and positive and negative predictive values for a d-dimer cut-off value of 8.6 mg/L were 100%, 82.5%, 30% and 100%, respectively, for symptomatic PE [25]. Meanwhile, Nakao et al. [24] showed that the plasma d-dimer cut-off value of 10 mg/L had a sensitivity and specificity of 94.4% and 90%, respectively, for the development of VTE among patients undergoing TKA. We utilized the various cut-off values mentioned above, and the proportions of subjects exceeding the three cut-off values were all significantly higher in the conventional group than in the navigation-assisted TKA group.

The differential extent of systemic embolization between navigation-assisted TKA and conventional TKA has been reported previously. Ooi et al. evaluated the degree, duration and size of the embolic shower using a transesophageal echocardiography probe after navigation-assisted and conventional TKA and demonstrated significant differences in the size of the emboli and the Mayo Clinic score between groups. There were also significant between-group differences in the pulse oximetry oxygen saturation and heart rate [21]. Kalairajah et al. undertook a prospective, randomized study using a transcranial Doppler device to compare the extent of cranial embolization in navigation-assisted (*n* = 14) and conventional TKA patients (*n* = 10). Doppler signals were obtained in 14 patients in the navigation-assisted group and nine (90%) in the conventional group, in whom high-intensity signals were detected in seven patients undergoing navigation-assisted TKA (50%) and all participants receiving conventional TKA. In the navigation-assisted group, no patient had more than two detectable emboli, with a mean of 0.64 (standard deviation: 0.74). In the conventional group, the number of emboli ranged from 1 to 43, and six patients had more than two detectable emboli, with a mean of 10.7 (standard deviation: 13.5). The between-group difference was highly significant (*p* < 0.001). The authors thus concluded that navigation-assisted TKA, when compared with the conventional technique, significantly reduces systemic emboli as detected by transcranial Doppler ultrasonography [22]. In keeping with the sonographic findings, our study showed that the plasma d-dimer level and the surge in the plasma d-dimer level 24 h after TKA were significantly higher in the conventional group. These combined findings cannot be extrapolated to conclude that navigation-assisted TKA leads to fewer postoperative thromboembolic complications; however, the correlations of the postoperative plasma d-dimer level with sonographically detected emboli and the occurrence of VTE warrant further study with a larger population for validation.

In addition to the correlation with thromboembolic events, Shahi et al. [28] suggested that a higher plasma d-dimer level is associated with a higher risk of periprosthetic joint infection (PJI). Although the causal relationship between a higher postoperative plasma d-dimer level and a higher risk of PJI is not clear, the findings suggested that the interpretation of a higher postoperative plasma d-dimer level among subjects undergoing conventional TKA should not be limited to the perspective of thromboembolic activity.

The limitations of this study included non-randomization and a relatively small number of patients. Although measurement of thromboembolic markers alone is not sufficient to diagnose VTE, it does represent activation of coagulation and the thrombotic cascade. According to previous studies, the plasma d-dimer level is correlated with the occurrence of VTE, as well as PJI. Although the lower perioperative plasma d-dimer level observed in the navigation-assisted TKA group could not be extrapolated to the conclusion that the incidences of VTE and PJI were lower in the navigation-assisted TKA group, our study may allow orthopedic surgeons to reflect upon the possibility of differential risks of VTE and PJI between navigation-assisted and conventional TKA techniques and offers important pilot findings that will inform subsequent larger-scale studies to compare the incidences of VTE and PJI between navigation-assisted TKA and conventional TKA.

## Conclusions

In our study, we demonstrated a lower plasma d-dimer level as well as a milder surge in the plasma d-dimer level in patients receiving navigation-assisted TKA as compared with those undergoing conventional TKA 24 h after surgery. The proportions of subjects exceeding the plasma d-dimer level cut-off values of 7.5, 8.6 and 10 mg/L 24 h after TKA were all significantly higher in the conventional group than in the navigation-assisted group. These findings may supplement the known benefits of navigation-assisted TKA.

## Methods

The study was approved by the Institutional Review Board of Chang Gung Memorial Hospital (IRB number: 100-0038A3) and registered on the ClinicalTrials.gov website (registration number: NCT02206321). Patients undergoing TKA surgery performed by the two senior authors (JYK and CJW) between September 2011 and November 2013 were recruited. The patients were self-separated according to their clinic registration to the two senior surgeons. One surgeon (JYK) performed navigation-assisted TKA, and the other surgeon (CJW) performed conventional TKA; the two surgeons had performed more than one thousand navigation-assisted or conventional TKA surgeries, respectively. Patients aged > 85 years, those with autoimmune diseases, active infection, malignancy, end-stage renal disease or a previous history of VTE, were excluded from the study. Patients who had undergone previous surgeries, such as high tibia osteotomy or fracture repair surgeries, on the same knee were also excluded. All participants provided valid informed consent and were aware of their surgical allocation prior to the operation, as the participants undergoing navigation-assisted TKA required four additional small incisions for tracker pinning.

Navigation-assisted TKA was performed with the aid of a CT-free navigation system (Vector Vision, Brain Lab, Heimstetten, Germany). Both femur cutting and tibia cutting were performed in an extramedullary manner in the navigation-assisted group, whereas intramedullary guided femur cutting was performed in the conventional TKA group.

Blood samples were obtained prior to and at 24 and 72 h after TKA surgery and were sent to the central laboratory in tubes loaded with sodium citrate for further analysis. The plasma d-dimer level was measured using a microlatex immunoturbidity technique (Dade Behring, Marburg, Germany), which employed a monoclonal antibody to detect only cross-linked d-dimer fragments, while fibrinogen was measured using a scattered light detection method.

Each patient began quadriceps exercise and continuous passive range of motion exercise 24 h after surgery, and most patients started off-bed ambulation on the same day. Oral aspirin at 500 mg per day was given for DVT prophylaxis. However, in patients with morbid obesity, lower leg swelling (Homan sign positive), or a plasma d-dimer level greater than 5 mg/L, oral rivaroxaban or subcutaneous enoxaparin was prescribed [19]. Venography or ultrasonography was arranged if DVT was clinically suspected, whereas chest CT angiography or lung perfusion scans were scheduled if PE was suspected. For patients without clinically suspected DVT, early off-bed ambulation and vigorous physiotherapy programs were encouraged as a mechanical VTE prophylaxis.

A packed red blood cell transfusion was given if the postoperative level of Hb < 7.0 g/dL or Hb < 8.0 g/dL with symptoms such as dizziness or generalized weakness. An oral iron supplement was prescribed for patients with asymptomatic anemia. Clinical assessments for DVT were performed daily until discharge and at regular outpatient follow-ups. Patients with postoperative comorbidities were advised to visit the Emergency Department.

The minimum sample size required for the navigation-assisted and control groups was determined using G*Power 3.1.9.2 software (http://www.gpower.hhu.de/en.html). The priori power calculation used a two-tailed Wilcoxon signed-rank test to calculate a sample size of at least 27 for each group (calculated effect size: 0.8; *α* level: 0.05; power: 80%; allocation ratio: 1) [22].

The data are presented as median values with lower and upper quartiles. Categorical variables were compared using the chi-square test, and the Mann–Whitney *U* test was employed to compare differences between groups. The Friedman test was used for repeated measures analysis of repeated within-group comparisons for continuous variables, and the Wilcoxon signed-rank test was used for post hoc analysis. All statistical analyses were performed using SPSS software, and a *p*-value of < 0.05 was considered to indicate statistical significance [2, 29, 30].

## Data Availability

The datasets used and/or analyzed during the current study are available from the corresponding author on reasonable request.

## Abbreviations

AAOS: American Academy of Orthopedic Surgeons
BMI: body mass index
CAD: coronary artery disease
PE: pulmonary embolism
VTE: venous thromboembolism
TKA: total knee arthroplasty
